# Fast monitoring of epileptic seizures using recurrence time statistics of electroencephalography

**DOI:** 10.3389/fncom.2013.00122

**Published:** 2013-10-01

**Authors:** Jianbo Gao, Jing Hu

**Affiliations:** ^1^Institute of Complexity Science and Big Data Technology, Guangxi UniversityNanning, China; ^2^PMB Intelligence LLCSunnyvale, CA, USA

**Keywords:** EEG, recurrence time, seizure detection, seizure propagation, brain complexity

## Abstract

Epilepsy is a relatively common brain disorder which may be very debilitating. Currently, determination of epileptic seizures often involves tedious, time-consuming visual inspection of electroencephalography (EEG) data by medical experts. To better monitor seizures and make medications more effective, we propose a recurrence time based approach to characterize brain electrical activity. Recurrence times have a number of distinguished properties that make it very effective for forewarning epileptic seizures as well as studying propagation of seizures: (1) recurrence times amount to periods of periodic signals, (2) recurrence times are closely related to information dimension, Lyapunov exponent, and Kolmogorov entropy of chaotic signals, (3) recurrence times embody Shannon and Renyi entropies of random fields, and (4) recurrence times can readily detect bifurcation-like transitions in dynamical systems. In particular, property (4) dictates that unlike many other non-linear methods, recurrence time method does not require the EEG data be chaotic and/or stationary. Moreover, the method only contains a few parameters that are largely signal-independent, and hence, is very easy to use. The method is also very fast—it is fast enough to on-line process multi-channel EEG data with a typical PC. Therefore, it has the potential to be an excellent candidate for real-time monitoring of epileptic seizures in a clinical setting.

## 1. Introduction

Epilepsy is a relatively common brain disorder which may be very debilitating. It affects approximately 1% of the world population (Jallon, 1997) and three million people in the United States alone. It is characterized by intermittent seizures. During a seizure, the normal activity of the central nervous system is disrupted. The concrete symptoms include abnormal running/bouncing fits, clonus of face and forelimbs, or tonic rearing movement as well as simultaneous occurrence of transient EEG signals such as spikes, spike and slow wave complexes or rhythmic slow wave bursts. Clinical effects may include motor, sensory, affective, cognitive, automatic and physical symptomatology. Although epilepsy can be treated effectively in many instances, severe side effects may result from constant medication. Even worse, some patients may become drug-resistant not long after treatment. To make medications more effective, timely detection of seizure is very important.

In the past several decades, considerable efforts have been made to detect/predict seizures through non-linear analysis of EEGs (Kanz and Schreiber, 1997; Gao et al., 2007). Representative non-linear methods proposed for seizure prediction/detection include approaches based on correlation dimension (Lehnertz and Elger, 1995, 1997; Martinerie et al., 1998; Aschenbrenner-Scheibe et al., 2003), Kolmogorov entropy (van Drongelen et al., 2003), permutation entropy (Cao et al., 2004), short time largest Lyapunov exponent (STLmax) (Iasemidis et al., 1990; Lai et al., 2003), dissimilarity measures (Protopopescu et al., 2001; Quyen et al., 2001), long-range-correlation (Hwa and Ferree, 2002; Gao et al., 2006b, 2007, 2011b; Valencia et al., 2008), power-law sensitivity to initial conditions (Gao et al., 2005b), scale-dependent Lyapunov exponent (SDLE) (Gao et al., 2006a, 2012a,b), and synthesis of linear/non-linear methods by using neural networks (Adeli et al., 2007). Readers interested in “what is epilepsy, where, when, and why (how) do seizures occur?” are referred to the April, 2007 issue of *Journal of Clinical Neurophysiology*.

Note that most of the proposed methods assume that EEG signals are chaotic and stationary. As a result, they tend to have performances that are signal- and patient-dependent due to the noisy and non-stationary nature of the EEG within and across patients. In addition, they are computationally expensive. Consequentially, studies of epilepsy still heavily involve visual inspection of multi-channel EEG signals by medical experts. Visual inspection of long (e.g., tens of hours or days) EEG data is, however, tedious, time-consuming, and in-efficient. Therefore, it is important to develop new non-linear seizure monitoring approaches.

In this paper, we explore recurrence time based analysis of EEG (Gao, 1999, 2001; Gao and Cai, 2000; Gao et al., 2003), with the goal of potentially on-line monitoring the occurrence and propagation of seizures. The method does not assume that the underlying dynamics of EEGs be chaotic or stationary. More importantly, it has been tested to be able to readily detect very subtle changes in signals (Gao, 2001; Gao et al., 2003).

**Figure 1:**
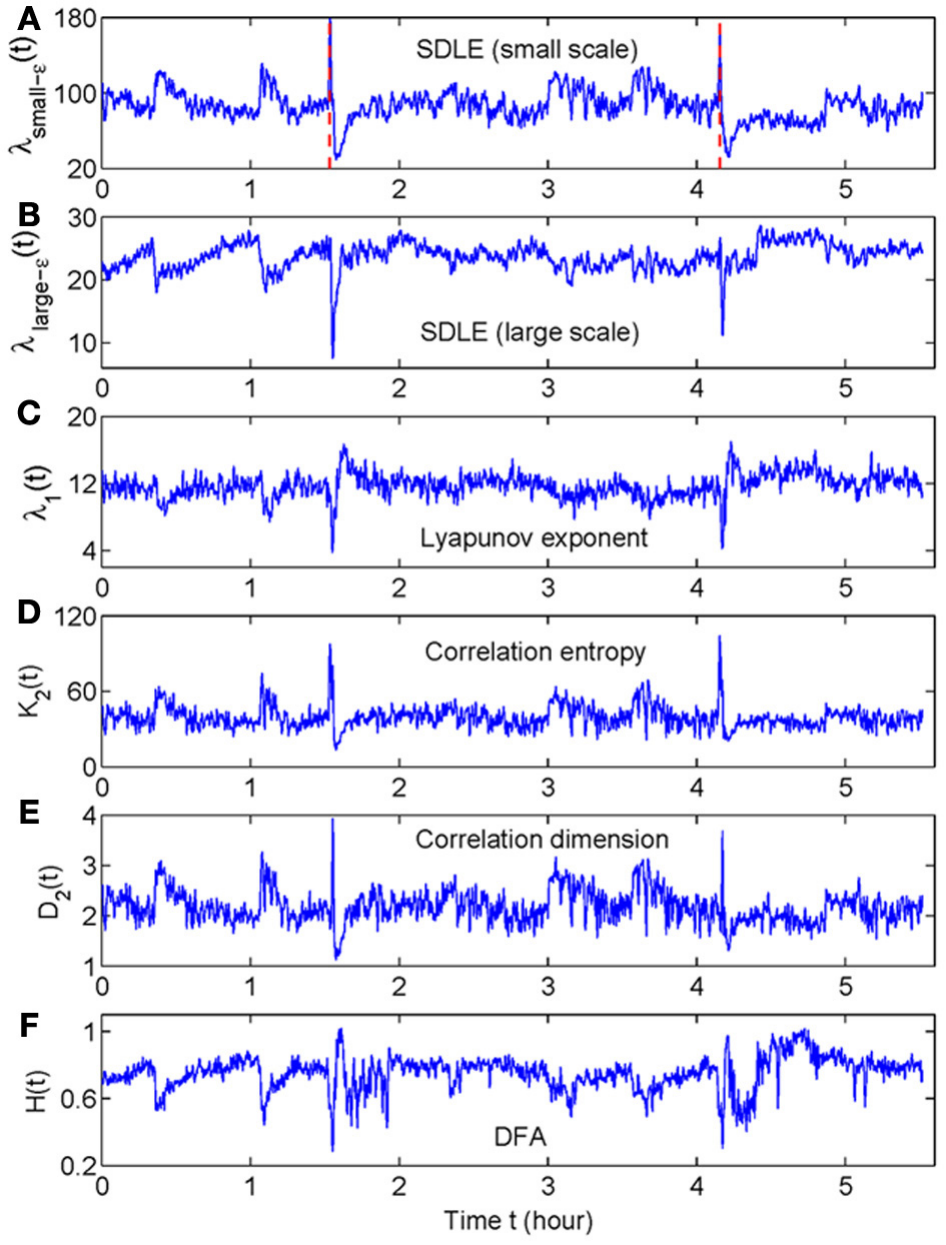
**The variation with time of (A) λ_small−ε_, (B) λ_large−ε_, (C) Lyapunov exponent, (D) correlation entropy, (E) correlation dimension, and (F) the Hurst parameter obtained using DFA.** Adapted from Gao et al. (2011a).

When developing a new method, it is important to compare its performance with that of existing methods. For seizure detection, such a task has been greatly simplified by our recent studies (Gao et al., 2011a, 2012a). By comparing seizure detection using a variety of complexity measures from deterministic chaos theory, random fractal theory, and information theory, we have found that the variations of those complexity measures with time have two patterns—either similar or reciprocal (Gao et al., 2011a). More importantly, we have gained fundamental understanding about the connections among different complexity measures through a new multiscale complexity measure, the SDLE. These results are recapitulated in Figure 1. While we leave the details to our prior works (Gao et al., 2006a, 2007, 2012a,b), these results suggest that it would be sufficient for us to compare the performance of the recurrence time based method for seizure detection with the performance of any of the existing complexity measures. Since some of the EEG data examined here had also been analyzed by the STLmax method and documented results exist, we shall compare our recurrence time method with the STLmax method. We shall show that the recurrence time method is both more accurate and faster than the STLmax method in detecting seizures from EEG.

The remainder of the paper is organized as follows. In section 2, we describe the data used here and the recurrence time method and the STLmax method for seizure detection. In section 3, we compare the performance of the recurrence time and STLmax method for seizure detection, as well as study seizure propagation. In section 4, we make a few concluding remarks.

## 2. Materials and methods

In this section, we first describe EEG data used here, then describe the recurrence time method and the short-time Lyapunov exponent (STLmax) method.

### 2.1. Data

**Figure 2:**
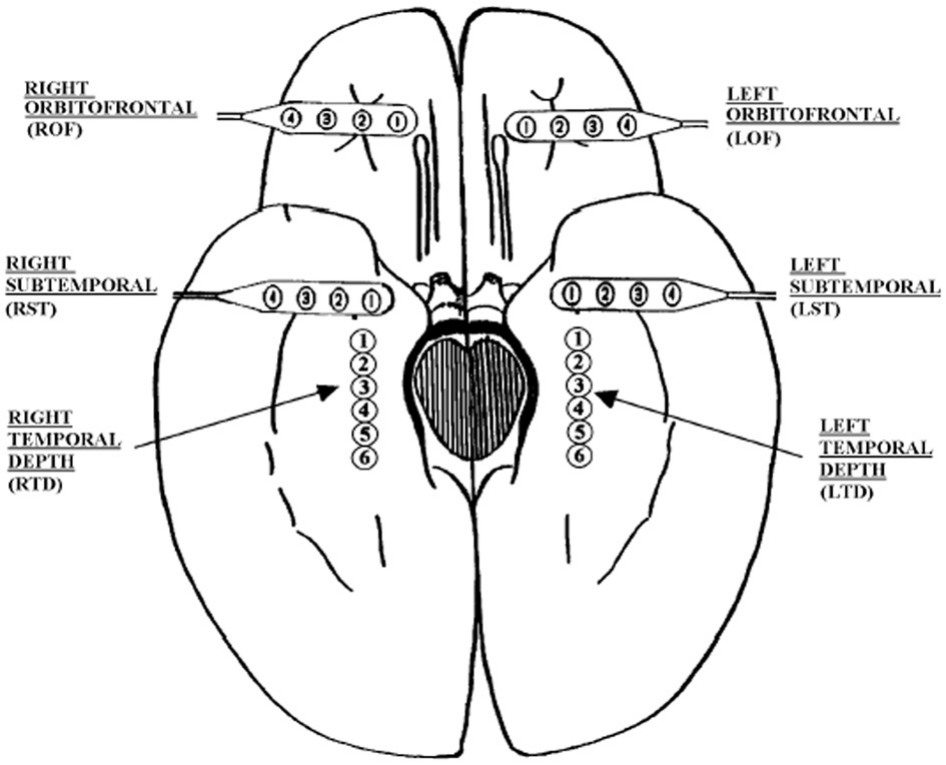
**Schematic diagram of the depth and subdural electrode placement.** This view from the inferior aspect of the brain shows the approximate location of depth electrodes, oriented along the anterior–posterior plane in the hippocampi (RTD, right temporal depth; LTD, left temporal depth), and subdural electrodes located beneath the orbitofrontal and subtemporal cortical surfaces (ROF, right orbitoftrontal; LOF, left orbitofrontal; RST, right subtemporal; LST, left subtemporal).

The EEG signals analyzed here are human EEG. They were recorded intracranially with approved clinical equipment by the Shands hospital at the University of Florida, with a sampling frequency of 200 Hz. Figure 2 shows our typical 28 electrode montage used for subdural and depth recordings.

Intracranial EEG is also called depth EEG, and is considered less contaminated by noise or motion artifacts. However, the clinical equipment used to measure the data has a pre-set, unadjustable maximal amplitude, which is around 5300 μV. This causes clipping of the signals when the signal amplitude is higher than this threshold. This is often the case during seizure episodes, especially for certain electrodes. To a certain extent, clipping complicates seizure detection, since certain seizure signatures may not be captured by the measuring equipment. However, we did not apply any filtering or conditioning methods to preprocess the raw EEG signals when we use our recurrence time method. The good results presented below thus suggest that the method is very reliable.

Altogether we have data of seven patients. The total duration of the measurement for each patient was up to about 3 days, as shown in the 2nd column of Table 1. There were only one or a few seizures for some patients while there were several tens of seizures for some other patients, as shown in the 3rd column of Table 1. Some of the seizures were considered subclinical, i.e., not manifested in the EEG signals. Sometimes the EEG signals may contain signatures distinctly different from background non-seizure signals, due to, for example, the fact that the patient may be eating food, drinking, etc. These non-seizure signatures typically may also be picked up by a seizure monitoring method. In this study, we shall focus on the behavior of the recurrence time and STLmax method in detecting seizures using only three channels EEG data without any preprocessing. As we shall see later, reliable decisions can be made based on single channel EEG data. There appears to be no need to combine multiple channels data.

**Table 1:** **Performance of the *T*_2_ and the STLmax method for seven patients' data**.

**Data set**	**Length (hours)**	**Total number of seizures**	**STLmax performance**		**T2 performance**	
				
			**Sensitivity (%)**	**False alarm per hour**	**Sensitivity (%)**	**False alarm per hour**
			**Overall: 74%**	**Mean: 0.05**	**Overall: 83%**	**Mean: < 0.01**
P92	35	7	100	0.09	100	0.00
P93	64	23	78	0.02	78	0.02
P148	76	17	58	0.07	76	0.00
P185	47	19	73	0.02	89	0.04
P40	5.3	1	100	0.00	100	0.00
P256	4.5	1	100	0.00	100	0.00
P130	5.7	2	50	0.18	100	0.00

*The total number of seizures was determined by examining clinical symptons and all 28 channel video-EEG data by medical experts. Note the five missed seizures for patient P93 are all subclinical seizures, whose information does not appear to be reflected by the EEG dynamics*.

### 2.2. Recurrence time based method for seizure detection

The method involves first partitioning a long EEG signal into (overlapping or non-overlapping) blocks of data sets of short length *k*, and compute the so-called mean recurrence time of the 2nd type, ${\bar{T}}_{2}(r)$, for each data subset. For non-stationary and transient time series, it has been found (Gao, 1999, 2001; Gao and Cai, 2000; Gao et al., 2003) that ${\bar{T}}_{2}(r)$ will be different for different blocks of data subsets.

**Figure 3:**
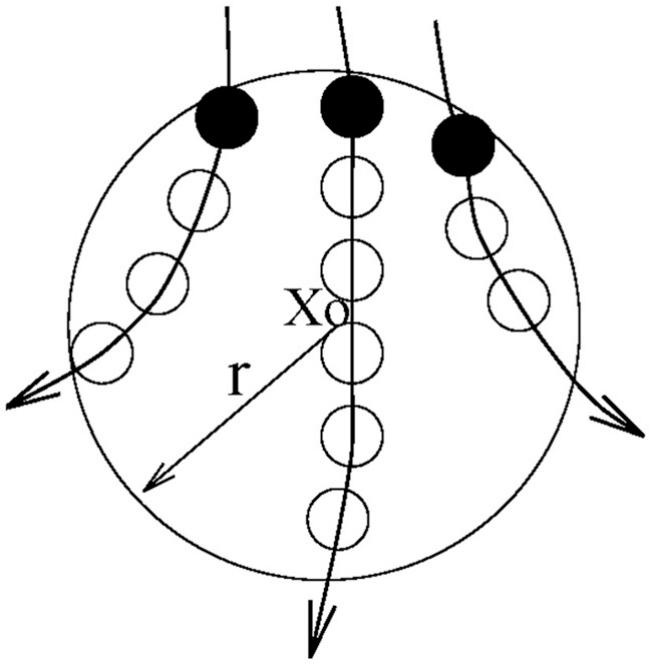
**A schematic showing the recurrence points of the second type (solid circles) and the sojourn points (open circles) in *B*_*r*_(*X*_0_)**.

Let us first define the recurrence time of the 2nd type. Suppose we are given a scalar time series {*x*(*i*), *i* = 1, 2, …}. We first construct vectors of the form: *X*_*i*_ = [*x*(*i*), *x*(*i* + *L*), …, *x*(*i* + (*m* − 1)*L*)], with *m* being the embedding dimension and *L* the delay time (Packard et al., 1980; Takens, 1981; Sauer et al., 1991). {*X*_*i*_, *i* = 1, 2, …, *N*} then represents certain trajectory in a *m*-dimensional space. Next, we arbitrarily choose a reference point *X*_0_ on the reconstructed trajectory, and consider recurrences to its neighborhood of radius *r*: *B*_*r*_(*X*_0_) = {*X*: ∥*X* − *X*_0_∥ ≤ *r*}. The recurrence points of the 2nd type are defined as the set of points comprised of the first trajectory point getting inside the neighborhood from outside. These are denoted as the dark solid circles in Figure 3. The trajectory may stay inside the neighborhood for a while, thus generating a sequence of points, as designated by open circles in Figure 3. These are called sojourn points (Gao, 1999). It is clear that there will be more such points when the size of the neighborhood gets larger as well as when the trajectory is sampled more densely. The summation of the recurrence points of the second kind and the sojourn points is called the recurrence points of the first kind. These are often called nearest neighbors of the reference point *X*_0_, and have been used by all other chaos theory-based non-linear methods.

Let us be more precise mathematically. We denote the recurrence points of the 1st type by *S*_1_ = {*X*_*t*_1__, *X*_*t*_2__, …, *X*_*t*_*i*__ …}, and the corresponding Poincare recurrence time of the 1st type by {*T*_1_(*i*) = *t*_*i* + 1_ − *t*_*i*_, *i* = 1, 2, …}. Note the time is computed based on successive returns, not based on the returning points and the reference point. Also note *T*_1_(*i*) may be 1 (for continuous time systems, this means one unit of the sampling time), for some *i*. This occurs when there are at least one sojourn point. Existence of such points makes further quantitative analysis difficult. Thus, we remove the sojourn points from the set *S*_1_ (which can be easily achieved by monitoring whether the recurrence times of the first type are one or not). Let us denote the remaining set by *S*_2_ = {*X*_*t′*_1__, *X*_*t′*_2__, …, *X*_*t′*_*i*__ …}. *S*_2_ then defines a time sequence {*T*_2_(*i*) = *t′*_*i* + 1_ − *t′*_*i*_, *i* = 1, 2, …}. These are called the recurrence times of the 2nd type.

*T*_2_(*i*) has a number of interesting properties: (1) For periodic motions, so long as the size of the neighborhood is not too large, *T*_2_(*i*) accurately estimates the period of the motion. (2) For discrete sequences, the entire Renyi entropy spectrum can be computed from the moments of *T*_2_ (Gao et al., 2005a). (3) For chaotic motions, *T*_2_(*i*) is closely related to the Lyapunov exponent, and hence, Kolmogorov entropy (Gao and Cai, 2000). (4) For chaotic motions, *T*_2_(*i*) is related to the information dimension *d*_1_ by a simple scaling law (Gao, 1999; Gao et al., 2003),

(1) $$T_{2}(r)\sim r^{d_{1}-\alpha },$$

where α takes on value 0 or 1, depending on whether the sojourn points form very few isolated points inside the neighborhood *B*_*r*_(*X*_0_), thus contribute dimension 0, or form a smooth curve inside *B*_*r*_(*X*_0_), thus contribute dimension 1. These properties make the recurrence time based method very versatile and powerful in detecting signal transitions.

**Figure 4:**
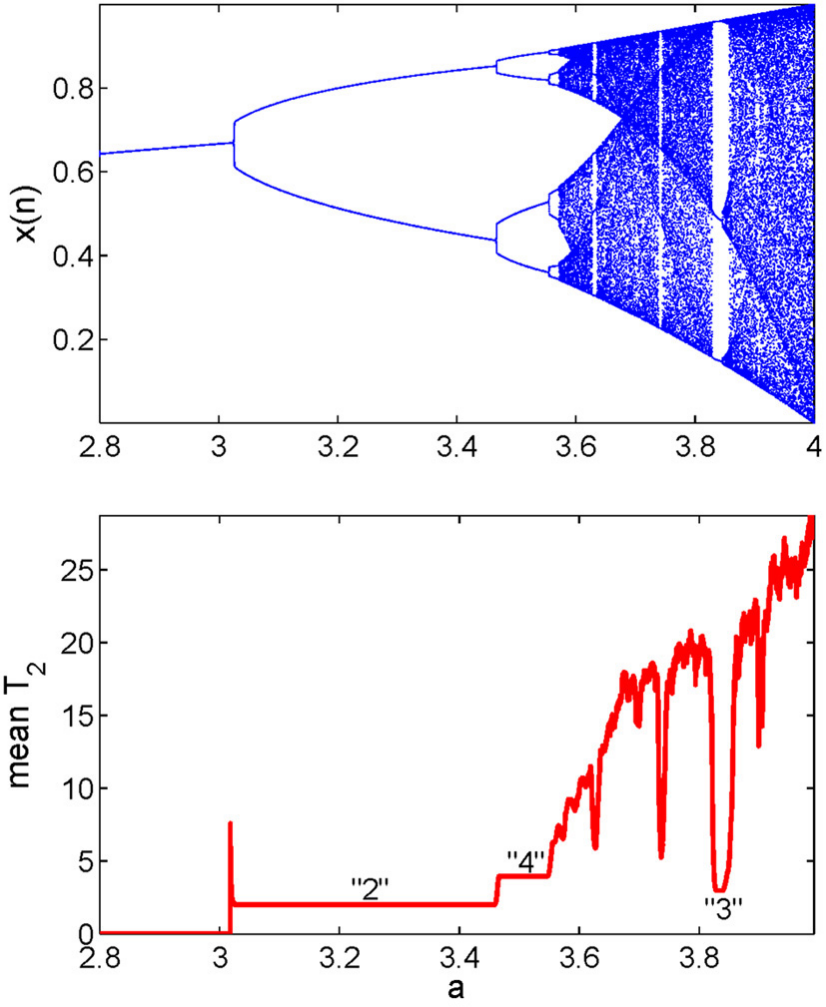
**State transitions in the transient logistic map**.

We now explain how the mean recurrence time of the 2nd type can be computed. We simply evaluate this quantity for every reference point in a window, then take the mean of those times. Such calculation is carried out for all the data subsets, resulting in a curve which describes how ${\bar{T}}_{2}(r)$ varies with time. It has been observed (Gao, 1999, 2001; Gao and Cai, 2000; Gao et al., 2003) that the variations of ${\bar{T}}_{2}(r)$ coincide very well with sudden changes in the signal dynamics, such as bifurcations or transitions from regular motions to chaotic motions in non-stationary data, and vise versa. An example is shown in Figure 4 using the transient logistic map described by

(2) $$x(n+1)=a(n)x(n)[1-x(n)],a(n)=a(n-1)+10^{-5}$$

We observe from Figure 4 that the method not only detects all the bifurcations in the signal, but also gives the exact periods of periodic signals. Note that some changes in a signal may be difficult to detect visually (Gao, 2001).

Since there are altogether four parameters involved, namely, the embedding dimension *m* and delay time *L*, the window length *k* for the data subsets, and the neighborhood size *r*, how shall we select them properly? To better illustrate the ideas, we postpone the discussion to section 3.1.1.

### 2.3. STLmax method for seizure detection

The basic idea is to compute the largest positive Lyapunov exponent for each window's EEG signal using the Wolf et al.'s algorithm (Wolf et al., 1985) or its simple variants. Therefore, it is sufficient to describe the Wolf et al.'s algorithm (Wolf et al., 1985) and point out how it can be modified.

To apply the Wolf et al.'s algorithm (Wolf et al., 1985), one selects a reference trajectory and follows the divergence of its neighboring trajectory from it. Denote the reference and the neighboring trajectories by *X*_*i*_ = [*x*(*i*), *x*(*i* + *L*), …, *x*(*i* + (*m* − 1)*L*)], *X*_*j*_ = [*x*(*j*), *x*(*j* + *L*), …, *x*(*j* + (*m* − 1)*L*)], *i*, = 1, 2, …, *j* = *K*, *K* + 1, …, respectively. At the start of the time (which corresponds to *i* = 1), *X*_*K*_ is usually taken as the nearest neighbor of *X*_1_. That is, *j* = *K* minimizes the distance between *X*_*j*_ and *X*_1_. When time evolves, the distance between *X*_*i*_ and *X*_*j*_ also changes. Let the spacing between the two trajectories at time *t*_*i*_ and *t*_*i* + 1_ be *d*′_*i*_ and *d*_*i* + 1_, respectively. Assuming *d*_*i* + 1_ ~ *d*′_*i*_*e*^λ_1_ (*t*_*i* + 1_ − *t*_*i*_)^, the rate of divergence of the trajectory, λ_1_, over a time interval of *t*_*i* + 1_ − *t*_*i*_ is then

$$\frac{\ln(d_{i+1}/d_{i}^{\text{'}})}{t_{i+1}-t_{i}}.$$

To ensure that the separation between the two trajectories is always small, when *d*_*i* + 1_ exceeds certain threshold value, it has to be renormalized: a new point in the direction of the vector of *d*_*i* + 1_ is picked up so that *d*′_*i* + 1_ is very small compared to the size of the attractor. After *n* repetitions of stretching and renormalizing the spacing, one obtains the following formula:

(3) $$\begin{array}{l} \lambda_{1}=\sum_{i=1}^{n-1}\left[\frac{t_{i+1}-t_{i}}{\sum_{i=1}^{n-1}\left(t_{i+1}-t_{i}\right)}\right]\left[\frac{\text{In}(d_{i+1}/{d^{\prime }}_{i})}{t_{i+1}-t_{i}}\right]\\ \;=\frac{\sum_{i=1}^{n-1}\text{In}(d_{i+1}/{d^{\prime }}_{i})}{t_{n}-t_{1}}. \end{array}$$

Note that this algorithm assumes but does not verify exponential divergence. In fact, the algorithm can yield a positive value of λ_1_ for any type of noisy process so long as all the distances involved are small. The reason for this is that when *d*′_*i*_ is small, evolution would move *d*′_*i*_ to the most probable spacing, which is typically much larger than *d*′_*i*_. Then, *d*_*i* + 1_, being in the middle step of this evolution, will also be larger than *d*′_*i*_; therefore, a quantity calculated based on Equation (3) will be positive. This argument makes it clear that the algorithm cannot distinguish chaos from noise. In other words, even if the algorithm returns a positive λ_1_ from EEG data, one cannot conclude that the data are chaotic.

It is worth noting that in practice, to simplify implementation of the algorithm, one may replace the renormalization procedure described above by requiring that *d*′_*i* + 1_ is constructed whenever *t*_*i* + 1_ = *t*_*i*_ + *T*, where *T* is a small time interval. Such a procedure may be called periodic renormalization. In contrast, the original version of the algorithm is an aperiodic renormalization.

## 3. Results

### 3.1. Seizure detection using recurrence time method

As we pointed out earlier, the method contains four parameters: the embedding dimension *m* and delay time *L*, the window length *k* for the data subsets, and the neighborhood size *r*. In this subsection, we first discuss how to choose these four parameters properly. Then we evaluate the effectiveness of the method for detecting epileptic seizures. For ease of presentation, we assume that the data have been normalized to the unit interval [0, 1] before further analysis.

#### 3.1.1. Parameter selection

**Figure 5:**
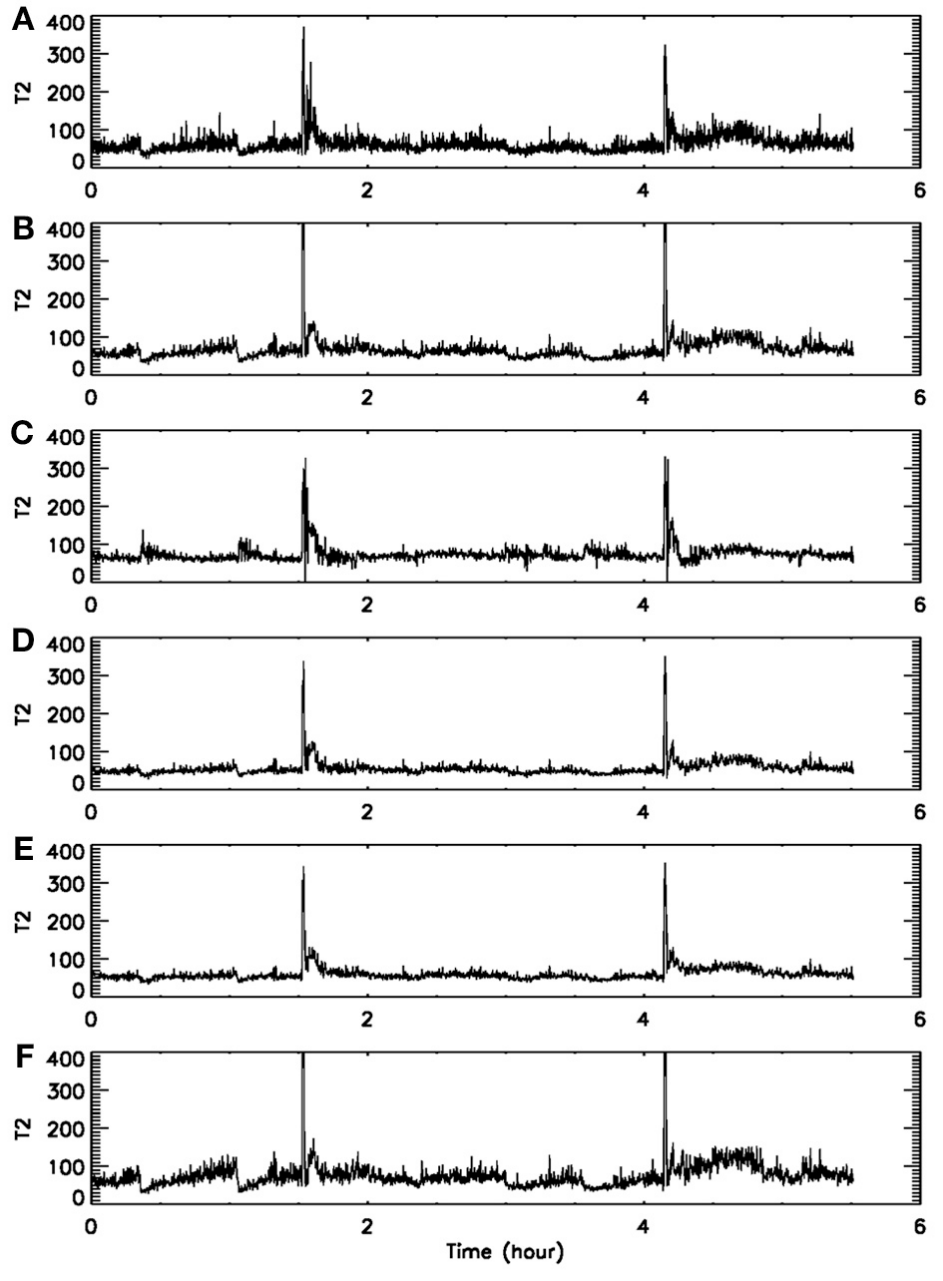
**Dependence of ${\bar{T}}_{2}$ on the parameters of the algorithm. (A–F)** Correspond to (*k*, *m*, *L*, *r*) = (1000, 4, 4, 2^−4^), (2000, 4, 4, 2^−4^), (2000, 4, 4, 2^−3^), (2000, 3, 4, 2^−4^), (2000, 4, 2, 2^−4^), and (2000, 4, 6, 2^−4^), respectively.

First, we consider the window length *k* for data subsets. Since our purpose is to find transitions in the signal dynamics, the data subset has to be small. In order to estimate the interesting statistics reliably, a rule of thumb is that so long as a data subset contains several periods of “oscillations”, it would be fine (assuming the motion defines certain periodicity-like time scales). For our EEG sampled with a frequency of 200 Hz, we have found that *k* in the range of 500–2000 are all fine. Figures 5A,B show two examples, for *k* = 1000 and 2000, respectively. Clearly, in both cases, the two seizures have been detected correctly.

Next, we consider the size *r* of the neighborhood. It can be readily appreciated that when *r* is large, there will be a lot of recurrences, while when *r* is small, recurrences will be rather rare. This means ${\bar{T}}_{2}(r)$ will be large for small *r* but small for large *r*. Such expectations have been extensively observed in practice. For EEG signals, we have found that although the values of ${\bar{T}}_{2}(r)$ may vary with *r*, the pattern of the variation basically remains the same for a wide range of *r*. Two examples are shown in Figures 5B,C, where *r* differs by a factor of 2. Our experience is that choice of this parameter is not very critical, in so far as seizure monitoring is concerned.

**Figure 6:**
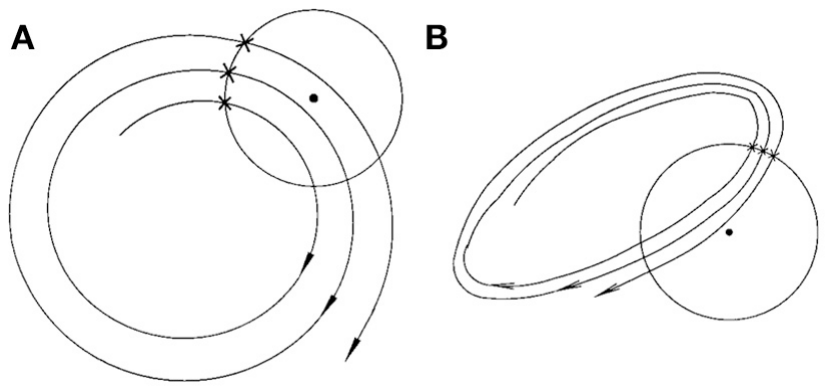
**A schematic showing the effect of embedding on the recurrence times of the second type. (A)** and **(B)** show examples of the reconstructed trajectory that is fairly uniform or less uniform, respectively.

Finally, we consider the embedding parameters. As is well known, the embedding parameters critically control the geometrical structure formed by the constructed vectors. Because of this feature, optimal embedding is a critical issue, especially when geometrical or dynamical quantities of the dynamics are concerned, such as the fractal dimension, Lyapunov exponents, and Kolmogorov entropy. For an in-depth discussion of this issue, we refer to Gao et al. (2007). Here, we wish to point out that the time scales associated with the motion are typically much less sensitive to the embedding parameters than the quantities such as the fractal dimension, Lyapunov exponents, and Kolmogorov entropy. To appreciate this feature, we have schematically shown in Figure 6 two different sets of embeddings. It is clear that the reconstructed trajectory shown in Figure 6A is fairly uniform, while that in Figure 6B is less so. One can readily conceive that when Figure 6B is further squeezed, the embedding quality is even worse. Judged by most optimal embedding criteria, the embedding shown in Figure 6A is considered a much better one than that shown in Figure 6B. However, it can be readily seen that ${\bar{T}}_{2}(r)$ for both Figures 6A,B are more or less the same. This means that the selection of *m* and *L* for computing ${\bar{T}}_{2}(r)$ is much less critical than that for computing other dynamical quantities. One good rule of thumb is that as long as the geometrical structure formed by the vectors are reasonably space-filling, the embedding is considered fine. Our experience with computing ${\bar{T}}_{2}(r)$ from EEG is that 3 ≤ *m* ≤ 6 are all fine, and with a sampling frequency of 200 Hz, *L* may be chosen 2–6. This discussion may be better appreciated by comparing Figures 5B,D–F, where four sets of (*m*, *L*) are illustrated. Clearly, all the parameter combinations have detected the two seizures accurately.

To summarize, the recurrence method is much less sensitive to the parameters when compared with other non-linear methods, where embedding and other parameters have to be chosen carefully, and have to be specifically adapted to each patient for good results. For our recurrence time method, however, we have used the same parameter combination (*k*, *m*, *L*, *r*) = (2000, 4, 4, 2^−4^) for all seven patients' data.

#### 3.1.2. Performance evaluation of the method

**Figure 7:**
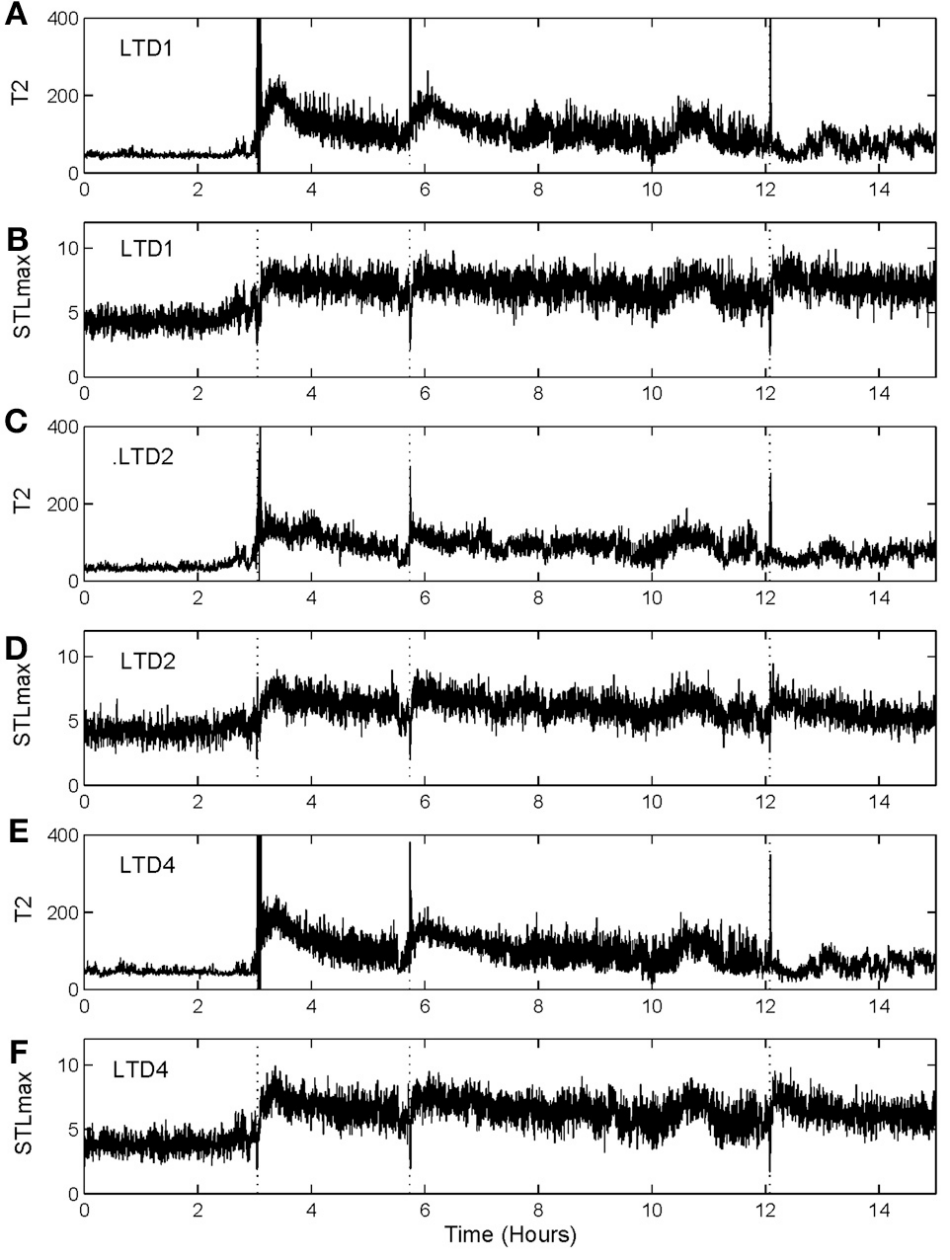
**${\bar{T}}_{2}(r)$ and STLmax vs. time curves for the EEG signals measured from three electrodes for a human patient. (A)** and **(B)** are for electrode LTD1, **(C)** and **(D)** for electrode LTD2, and **(E)** and **(F)** for electrode LTD4.

To illustrate the idea, we shall arbitrarily pick up three channels of EEG data,^1^ from one patient, and compare the patterns of variation of ${\bar{T}}_{2}(r)$ with that of STLmax. One typical result is shown in Figure 7. Vertical dotted lines indicate the seizure occurrence time determined by medical experts by viewing videotapes as well as the EEG signals. There are three seizures in Figure 7 during the period of time plotted. We observe that ${\bar{T}}_{2}(r)$ curves very cleanly and accurately detect all the seizures occurred. In fact, if one ignores the propagation-related slight timing difference (on the order of a few seconds up to 1 min; this will be further discussed later) among different electrodes, then most of the channels can be considered equivalent. In other words, decision can be based on single channel EEG data. This feature makes automatic detection of seizure by thresholding almost trivial. In contrast, the STLmax curves are much noisier than the ${\bar{T}}_{2}(r)$ curves. Although STLmax curves can be further post-processed to better reveal seizure information (Iasemidis et al., 2003), those features are still much weaker than those revealed by the recurrence time method.

To more systematically compare the performance of the two methods in detecting seizures, we have computed positive detection (or equivalently, sensitivity) and false alarm per hour for the two methods. Positive detection is defined as the ratio between the number of seizures correctly detected and the total number of seizures. The false alarm per hour is simply the number of falsely detected seizures divided by the total time period. Table 1 summarizes the results. Clearly, the recurrence time method is more accurate than the STLmax method. This accuracy becomes even more attractive if one notices that the recurrence time method only involves simple thresholding, while the STLmax method involves a lot of further analysis (Iasemidis et al., 2003).

#### 3.1.3. Computational cost

The recurrence time method is very fast. With an ordinary PC (CPU speed less than 2 GHz), computation of ${\bar{T}}_{2}(r)$ from one channel EEG data of duration 1 h with sampling frequency of 200 Hz takes about 1 min CPU time. Computation of STLmax, on the other hand, takes more than 10 min. Hence, the recurrence time based method is much faster than the STLmax method. In fact, even with an ordinary PC, one is able to process all 28 channels of 1-h EEG data in about half an hour, therefore, faster than the data being continuously collected. With a more powerful PC, of course, the speed becomes even faster. Such a speed implies that the method can be used to real-time on-line process continuously collected all channels of EEG data. From an engineering perspective, the fast computation of recurrence time statistics can be considered overwhelming.

### 3.2. Propagation of epileptic seizures in the brain

**Figure 8:**
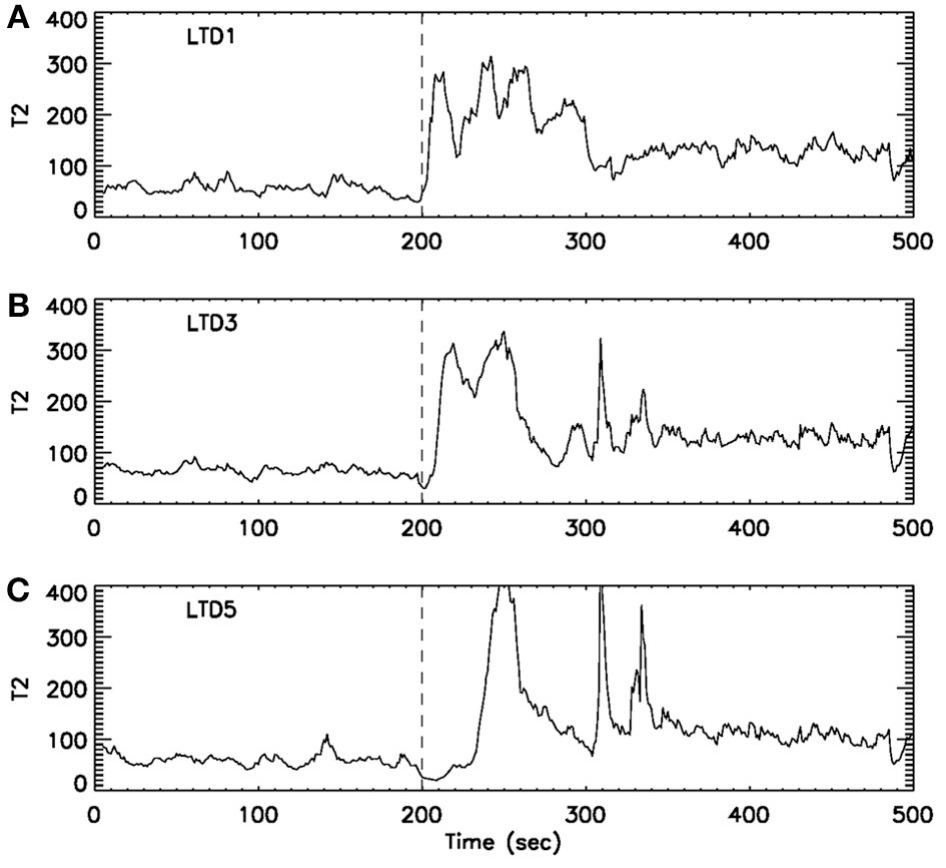
**${\bar{T}}_{2}(r)$ curves for EEG signals measured by three electrodes.** The dashed vertical line indicate the seizure starting position around 200 s. The seizure lasted for about 2 min. Note that from **(A)** LTD1 to **(B)** LTD3, the seizure activity is delayed about 10 s, while from **(A)** LTD1 to **(C)** LTD5, the seizure activity is delayed about 30–40 s.

Formation and propagation of epileptic seizures in the brain is an outstanding example of complex spatial-temporal pattern formations. One of the most desirable ways of studying these problems is to understand how and when information flows from one region of the system to other regions. To resolve this issue, it is critical to accurately providing timing information for interesting events occurring in the system. With the exact timing information, one can then use concepts such as cross correlation and cross spectrum, mutual information, or measures from chaos theory, such as related to cross recurrence plots, to more fully characterize the spatial–temporal patterns. Recurrence time method can effectively provide such a timing information. To illustrate this point, we have shown in Figure 8 an example of analysis of multi-channel EEG signals using the recurrence time method. For the specific seizure studied, it was known that the seizure occurred around 200 s, and lasted about 2 min. While the recurrence time method has accurately detected the seizure, we note that the seizure activity recorded by electrode LTD3 and LTD5 was about 10 and 40 s later than that indicated by electrode LTD1, respectively. Hence, the recurrence time method not only accurately detects the seizure, but also provides invaluable timing information for the development of the seizure.

## 4. Conclusions

Motivated by developing a non-linear method without the limitations of assuming that EEG signals are chaotic and stationary, we have proposed a recurrence time based approach to characterize brain electrical activity. The method is very easy to use, as it only contains a few parameters that are largely signal-independent. It very accurately detects epileptic seizures from EEG signals. Most critically, the method is very fast—it is fast enough to real-time on-line process multi-channel EEG data with a typical PC. Therefore, it has the potential to be an excellent candidate for real-time monitoring of epileptic seizures in a clinical setting.

The recurrence time method is also able to accurately give the timing information critical for understanding seizure propagation. Therefore, it may help characterize epilepsy type, lateralization and seizure classification (Holmes, 2008; Napolitano and Orriols, 2008; Plummer et al., 2008). To more thoroughly understand the capabilities of recurrence time method in characterizing seizure propagation, it would be desirable to combine recurrence time analysis of EEG with studies based on MEG and MRI exams.

### Conflict of interest statement

The authors declare that the research was conducted in the absence of any commercial or financial relationships that could be construed as a potential conflict of interest.

## Footnotes

^1^In fact, the three chosen channels EEG data may not correspond to where a seizure was localized. This further indicates the robustness of our method.
